# Overcoming the Limitation of Spin Statistics in Organic Light Emitting Diodes (OLEDs): Hot Exciton Mechanism and Its Characterization

**DOI:** 10.3390/ijms241512362

**Published:** 2023-08-02

**Authors:** Soo Wan Park, Dongwook Kim, Young Min Rhee

**Affiliations:** 1 Department of Chemistry, Korea Advanced Institute of Science and Technology (KAIST), Daejeon 34141, Republic of Korea; 2 Department of Chemistry, Kyonggi University, Suwon 16227, Republic of Korea

**Keywords:** OLED, hot exciton, triplet harvesting, hRISC, spin statistics

## Abstract

Triplet harvesting processes are essential for enhancing efficiencies of fluorescent organic light-emitting diodes. Besides more conventional thermally activated delayed fluorescence and triplet-triplet annihilation, the hot exciton mechanism has been recently noticed because it helps reduce the efficiency roll-off and improve device stability. Hot exciton materials enable the conversion of triplet excitons to singlet ones via reverse inter-system crossing from high-lying triplet states and thereby the depopulation of long-lived triplet excitons that are prone to chemical and/or efficiency degradation. Although their anti-Kasha characteristics have not been clearly explained, numerous molecules with behaviors assigned to the hot exciton mechanism have been reported. Indeed, the related developments appear to have just passed the stage of infancy now, and there will likely be more roles that computational elucidations can play. With this perspective in mind, we review some selected experimental studies on the mechanism and the related designs and then on computational studies. On the computational side, we examine what has been found and what is still missing with regard to properly understanding this interesting mechanism. We further discuss potential future points of computational interests toward aiming for eventually presenting in silico design guides.

## 1. Introduction

**Figure 1:**
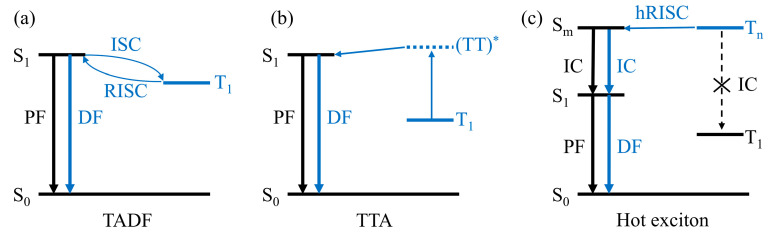
Schematics of triplet harvesting fluorescence: (**a**) thermally activated delayed fluorescence, (**b**) triplet-triplet annihilation, and (**c**) reverse inter-system crossing from hot exciton state.

Organic light-emitting diode (OLED) materials have been widely studied and utilized in recent years, as they are important for manufacturing flat-panel full-color displays. When powered on, OLEDs undergo four successive processes: charge injection, charge transport, charge recombination, and photon emission. The holes and electrons are injected from anodes and cathodes to hole- and electron-injection layers, respectively. They are carried to the emissive layer through the hole/electron transport layers. In the emissive layer (and/or at the interface between emissive and its neighboring layers), the two charge carriers combine to form excitons (charge recombination), and the excitons, through radiative decays, turn to photons toward the final emission. The exciton dynamics in various optoelectronic devices have been extensively studied recently [1,2,3,4,5]. In OLED, during the charge recombination, singlet and triplet excitons are formed in the ratio of 1:3 by the spin statistics [1]. Assuming the equal injection efficiencies and mobilities for both charges, the external quantum efficiency (EQE) is given by [6]

(1) $$\eta_{\mathrm{EQE}}=\gamma \phi_{\mathrm{PLQY}}\eta_{\mathrm{EUE}}\eta_{\mathrm{OC}}=\eta_{\mathrm{IQE}}\eta_{\mathrm{OC}}$$

where *γ* is the carrier recombination efficiency, *ϕ*_PLQY_ is the photoluminescence quantum yield (PLQY), $\eta_{\mathrm{EUE}}$ is the exciton utilization efficiency (EUE), and $\eta_{\mathrm{OC}}$ is the outcoupling efficiency. To utilize both types of excitons toward emission, phosphorescent emitters are often adopted. In red and green, iridium- or platinum-based emitters have been successfully used with an internal quantum efficiency (IQE) of near unity and EQEs of 20–25% [7]. On the other hand, blue phosphorescent emitters still have stability issues with undesirably short lifetimes [8,9]. With this persistent issue, developing fluorescent emitters that are free from rather expensive metals can be a very attractive alternative. However, the device performance with pure fluorescent emitters tends to be limited by the spin statistics, which leads to only ~25% of EUE. Therefore, somehow converting exciton spins from triplet to singlet will be highly desirable. Indeed, a series of such mechanisms have been employed. Namely, thermally activated delayed fluorescence (TADF) via reverse inter-system crossing (RISC), triplet-triplet annihilation (TTA), and hot exciton conversion, as schematically depicted in Figure 1, have been suggested as amenable strategies of converting triplet excitons into singlet ones.

Developing stable blue OLED materials is indeed a large challenge. High energy excitons can easily break chemical bonds, especially rather weak metal-containing bonds in phosphorescent OLED molecules. Nevertheless, recently, stable platinum-based and metal-free blue phosphorescent emitters have been reported [10,11,12,13]. At the same time, studies on stable fluorescent emitters are also being actively pursued. TADF emitters can yield nearly 100% IQE in the operating OLEDs [14], but their lifetimes tend not to be ideal together with somewhat large efficiency roll-offs caused by long-lived triplet excitons [15,16]. This is because high concentrations of triplet excitons induce exciton quenching processes into reducing emission efficiency, in addition to the molecular destruction mentioned above. Furthermore, traditional donor–acceptor (D–A) type emitters have a color purity issue. Using a color filter as a remedy unavoidably reduces EQE significantly. To circumvent this problem, a TADF-sensitized system dubbed as hyperfluorescence has been proposed [17,18,19]. TTA-based emitters have been reported to have good efficiency and lifetimes in practical applications [20,21]. However, they also have the efficiency roll-off problem, and their theoretical EUE is limited to 62.5%. In recent years, hot exciton materials are considered potentially superior candidates for blue OLED. Because the hot exciton materials can utilize fast hot reverse intersystem crossing (hRISC) processes from high-lying triplet states, they may suffer less from the stability or efficiency roll-off issues.

Naturally, theoretical and computational tools are useful for obtaining information that is not easily accessed with experimental means. For example, designing good TADF molecules often has to follow the path of finding molecules that have small S_1_–T_1_ energy gaps, large S_1_–T_1_ spin-orbit coupling (SOC), and large enough oscillator strengths in the S_1_ states. Knowing these factors can give useful guidelines, and computationally accessing them is nowadays not an excessively demanding task and is indeed adopted for screening purposes. Recently, vibronic effects on SOC or oscillator strengths have also been reported to be important [22,23,24,25,26,27], expanding the molecular space that needs to be explored. As noted in above, hot exciton materials are now considered as a good candidate for realizing high efficiency and stable longevity. Because the energy levels of singlet and triplet states and the SOC between them can also be used as guiding tools toward an efficient hRISC process, computational tools may prove themselves essential along the development. With this purpose in mind, we will review the triplet harvesting processes mentioned above. In particular, we will survey with eventual stresses the role of theoretical and computational studies in explaining hot exciton mechanism and possibly designing new materials for it. Computational studies can analyze the characteristics of states related to the hRISC process, so they may provide insights into the nature of hot exciton materials. Finally, we will discuss potential future works to better characterize high-lying states and their dynamics.

## 2. Overcoming Spin Statistics Limit in OLED 

### 2.1. Thermally Activated Delayed Fluorescence

Thermally activated delayed fluorescence (TADF) is one of the most attempted triplet harvesting processes and involves intersystem crossing (ISC) from the lowest triplet excited state (T_1_) to the lowest singlet one (S_1_), namely, reverse inter-system crossing (RISC). About a decade ago, Adachi and co-workers introduced the concept of TADF to OLED with a carbazole-phthalonitrile complex [28,29]. The maximum EQE of 1,2,3,5-tetrakis(carbazol-9-yl)-4,6-dicyanobenzene (4CzIPN) was more than 19%, which is still significantly higher than the EQEs of many other fluorescent emitters [29]. For effective RISC, the energy difference of S_1_ and T_1_ states, Δ*E*_ST_, should be comparable to the thermal energy *k*_B_*T*, and the spin-orbit coupling (SOC) between the two states should be large, preferably as large as at least several cm^−1^. 

The determination of TADF molecules is quite simple. First, Δ*E*_ST_ is very small, usually down to or smaller than 0.1 eV. Second, transient photoluminescence shows two decay characteristics, which are ns-scale prompt fluorescence and μs-scale delayed fluorescence. Third, the delayed fluorescence component is significantly reduced when temperature is lowered because RISC from T_1_ to S_1_ is driven by thermal energy. Fourth, their delayed fluorescence is abruptly quenched in the presence of oxygen molecules, demonstrating that it originates from the triplet state.

**Figure 2:**
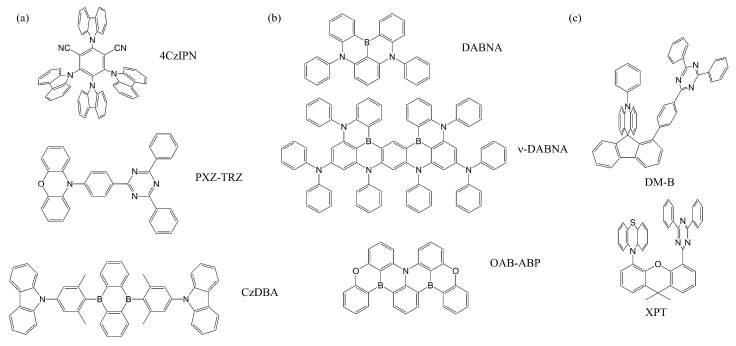
Exemplary TADF materials. (**a**) Donor–acceptor type, which spatially separates HOMO-LUMO in donor and acceptor units; (**b**) multiple resonance, which separates HOMO-LUMO in the same ring by the alternating orbital patterns; and (**c**) U-shaped molecules.

The energy difference has been known to be proportional to the spatial overlap between the highest occupied molecular orbital (HOMO) and the lowest unoccupied molecular orbital (LUMO) when the excitation characters of S_1_ and T_1_ states are simple HOMO to LUMO transitions [30,31,32]. Thus, designing D–A type molecules has been a main strategy for making TADF emitters (Figure 2a) [29,32,33]. Unfortunately, this D–A type strategy does not always work for two reasons. As mentioned above, decreasing HOMO–LUMO spatial overlap only works when both the S_1_ and the T_1_ excitation characters are donor-to-acceptor charge transfers (CTs). However, the T_1_ state prefers to have a local-excitation (LE) character in many molecules [34,35]. Therefore, Δ*E*_ST_ can become too large for a RISC process. On the other hand, when the S_1_ and T_1_ states are CT states, the SOC between the two states becomes negligible [36,37,38]. Therefore, designing TADF emitters that have small Δ*E*_ST_ and large SOC simultaneously is still challenging. Recent studies reported that spin-vibronic coupling can enhances the RISC process even in molecules with large Δ*E*_ST_ and/or small SOC [22,23,24,25,26]. This further complicates the design strategy but allows more molecules to become candidates.

Another issue in TADF emitters is the relatively weak and broad emission. The radiative decay rate is proportional to the square of the transition dipole moment (TDM) which is related to spatial overlap between hole and electron wavefunctions. Thus, D–A type molecules tend to have a small radiative decay rate compared to the nonradiative decay rate. Recent studies have shown that the vibronic coupling effect may enhance TDM in CT states compared to what is expected purely electronically [27], but even the enhanced value is still lower than the TDMs of many LE states. On the other hand, D–A type molecules usually show broad emission peaks originating from substantial structural relaxation. Namely, the donor and the acceptor units can be easily twisted, and the energy difference between S_1_ and S_0_ can vary in a wide range. For this reason, the color purity of a D–A type TADF emitter is low, and filtering is often needed, reducing the device’s efficiencies. In 2016, Hatakeyama and co-workers reported 5,9-diphenyl-5*H*,9*H*-[1,4]benzazaborino [2,3,4-*kl*]phenazaborine (DABNA), bearing the multiple resonance (MR) effect [39] that causes HOMO–LUMO spatial separation in the same rigid ring [40,41,42] (Figure 2b) [39,41,43]. With the rigid structure, the MR emitters have small Stokes shifts and thus narrow emission peaks. Modifications on D–A type molecules that add rigid bridges have also been studied [44,45,46,47]. For example, U-shaped molecules as shown in Figure 2c [44,48] can act as an intramolecular exciplex through space charge transfer such that the intramolecular π–π interaction restricts molecular motions and decelerates nonradiative decays [48]. In addition, CT characters are intensified in the T_1_ states of the U-shaped molecules due to the proximity between the D and A units, reducing Δ*E*_ST_ for better emission [45].

To solve the color purity issue, a TADF sensitizing scheme with hyperfluorescence was proposed by embedding a TADF sensitizer and a fluorescent emitter in the device [7]. Via RISC in the TADF sensitizer, triplet excitons convert to singlet excitons, which move to the fluorescent emitter via Förster resonance energy transfer (FRET). Dexter energy transfers (DET) of triplet excitons from the TADF sensitizer to the fluorescent emitter should be suppressed to prevent efficiency loss [17]. Recently, an MR TADF emitter was also used in hyperfluorescence devices instead of a fluorescent emitter [7,49]. Because the MR TADF emitter in this case can utilize triplet excitons, DET does not pose a challenge like in original hyperfluorescence systems, and this combination of a TADF sensitizer and an MR TADF emitter in a hyperfluorescence device can have low efficiency roll-off [49].

### 2.2. Triplet-Triplet Annihilation

**Figure 3:**
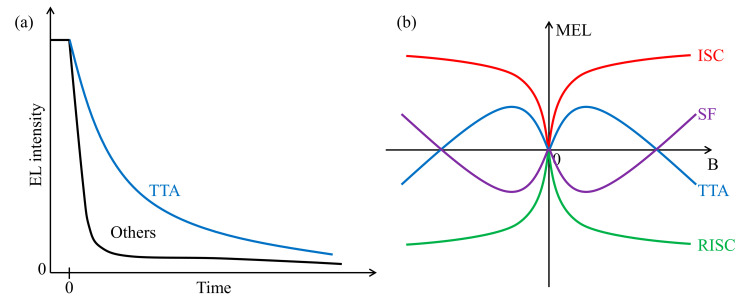
(**a**) Transient EL curves of TTA emitters and other fluorescent emitters. (**b**) Typical MEL patterns of ISC-dominant, RISC-dominant, TTA, and SF molecules as functions of magnetic field strength B.

Triplet-triplet annihilation (TTA) or triplet-triplet fusion (TTF) is a photon upconversion process where two triplet excitons (T_1_) merge into one exciton with twice the energy. The process can generate an emissive exciton if the fused state is spin singlet. However, the total number of excitons decreases with TTA, and thus in phosphorescent or TADF devices, it should be inhibited. In fluorescent devices, on the contrary, the TTA process is useful for increasing EUE. While it is still not easy to design blue phosphorescent or TADF emitters free from the stability and efficiency issues already described in the earlier section, TTA may in principle ameliorate the issues by providing a decay channel to triplet excitons. Nevertheless, because TTA is a bimolecular process, high triplet concentration is needed for effective TTA, which in turn can also lead to triplet-polaron quenching and/or triplet-singlet annihilation to reduce EUE. Therefore, TTA materials tend to suffer more from efficiency roll-offs. The transient electroluminescence (EL) [20] and magneto-electroluminescence (MEL) spectra [50] can be used to determine whether TTA is involved. In the case of transient EL, TTA devices typically exhibit delayed and long-lived luminescence after the devices are turned off, as depicted in Figure 3a [20,51]. In addition, TTA devices display unique MEL spectra; the MEL signal increases and then decreases as the external magnetic field increases, as sketched in Figure 3b [50,52].

The detailed mechanism of a TTA process is yet somewhat unclear, just like the details of singlet fission (SF), its inverse process, are not perfectly clear yet. During the process, two triplet excitons form an intermediate correlated triplet pair state (TT)*. To conserve the total angular momentum, the pair must be in either singlet (^1^(TT)*), triplet (^3^(TT)*), or quintet (^5^(TT)*) spin states. If the final state is determined by the spin statistics, they will form with the ratio of 1:3:5. After the generation, ^1^(TT)* splits to S_1_ and S_0_, and ^3^(TT)* splits to T*_n_* and S_0_. Because the lowest quintet excited state (Q_1_) energy is often too high to reach, ^5^(TT)* tends to return to original two T_1_. In addition, T*_n_* from ^3^(TT)* quickly relaxes to T_1_ according to Kasha’s rule. Therefore, if there are nine triplet pairs generated from eighteen T_1_ excitons, there will be one S_1_ and thirteen T_1_ remaining excitons. The upconversion efficiency ($\eta_{\mathrm{UC}}$) can thus be calculated as [53,54]

(2) $$\eta_{\mathrm{UC}}={\sum_{k=0}^{\infty }\frac{1}{18}\times \left(\frac{13}{18}\right)}^{k}=20\%$$

and the theoretical limit of EUE will be $\eta_{\mathrm{EUE}}=0.25+0.75\times 0.2=40\%$. If all upper triplet states are too high in energy for the condition where $E_{T_{2}}>2E_{T_{1}}>E_{S_{1}}$, then ^3^(TT)* also returns to the original two T_1_, and the theoretical limits of EUE and UC are, respectively, $\eta_{\mathrm{UC}}=50\%$ and $\eta_{\mathrm{EUE}}=0.25+0.75\times 0.5=62.5\%$.

**Figure 4:**
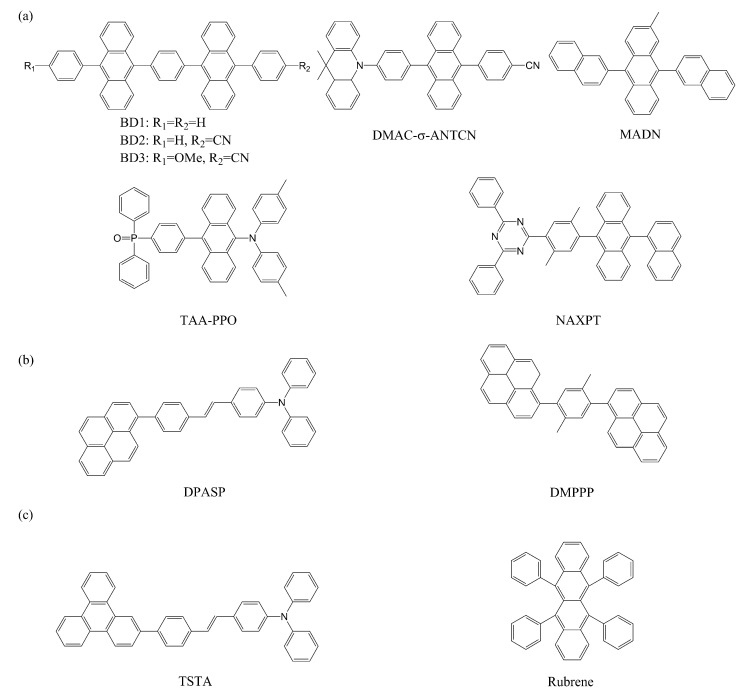
Exemplary (**a**) anthracene-based, (**b**) pyrene-based, and (**c**) other TTA materials. As shown in the figure, TTA materials usually contain polycyclic aromatic rings for satisfying the energy condition.

Most TTA materials feature polycyclic aromatic structures. Indeed, the majority of TTA materials contain anthracene units [55]. Twice the T_1_ energy (1.84 eV) of anthracene is slightly higher than its S_1_ energy (3.31 eV) and T_2_ energy (3.23 eV) [56], which renders the molecule effective for TTA, at least energetically. Thus, anthracene derivatives may act as good starting units toward designing new TTA materials, although non-anthracene polycyclic TTA molecules have also been reported [57,58,59]. Some representative TTA materials are shown in Figure 4 [54,58,59,60,61,62,63,64].

It may be unclear how a correlated triplet pair is produced, but it is possible to calculate the transition rate from the correlated triplet pair to its split singlet excited state (S_1_ + S_0_). Rate equations based on the Fermi golden rule and the Marcus theory have been used for this purpose [65,66]. The electronic coupling between the two states is often calculated by a fragment-based method [67]. These calculation results demonstrated that CT states should be included to reliably describe singlet fission or TTA processes [65,66,67] and that CT states may act as an intermediate state between the triplet pair and the split singlet excited state.

### 2.3. Hot Exciton Process

**Figure 5:**
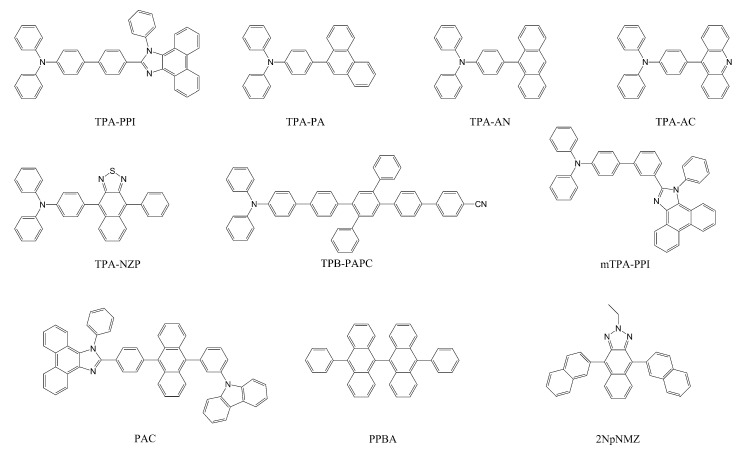
Exemplary hot exciton materials. The majority bears the donor–acceptor type structures.

Hot exciton process is another type of triplet harvesting mechanism via RISC. Unlike TADF, RISC in this case initiates not from T_1_ but from higher-lying triplet states (thus “hRISC”). Although the hot exciton process does not obey Kasha’s rule, numerous hot exciton materials have been reported [20,68,69,70,71,72,73,74,75,76]. A comprehensive review on the reported cases of hot exciton materials can be found elsewhere [20], and some representative molecules are shown in Figure 5 [51,77,78,79,80,81,82,83] with their photophysical properties summarized in Table 1. Ideally, hot exciton materials have several advantages. Because triplet excitons can be rapidly depopulated via hRISC, there will be a much smaller chance of having long-lived T_1_ excitons, significantly improving device stability and efficiency at high exciton concentrations. In addition, the S_1_ states of hot exciton molecules do not need to have dominant CT characters like in TADF molecules. Often, the S_1_ states are reported to have LE or hybridized local and charge-transfer (HLCT) characters. When LE character is intensified in the S_1_ state, hot exciton materials can exhibit high emission efficiency. These merits have raised many research interests in recent years, and in the next section, we will focus on topics related to the hot exciton process in more detail, including a brief history of research on the hot exciton process. We will also discuss experimental evidence reported in the literature, as well as computational aspects regarding the process itself and its design applications.

## 3. Accounts on Hot Exciton Processes

### 3.1. Hybridized Local and Charge-Transfer States

**Figure 6:**
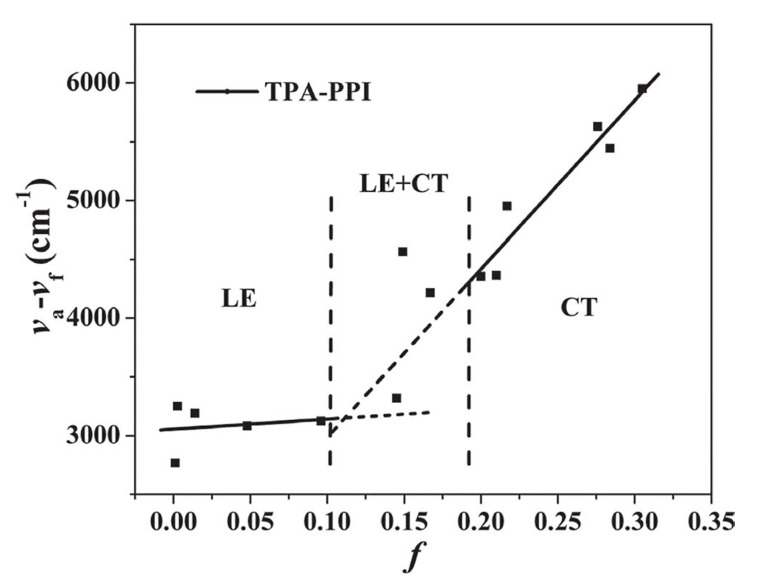
Linear correlation between the orientation polarization (*f*) of solvent media and the Stokes shift (*ν*_a_−*ν*_f_) for TPA-PPI. The dashed lines are the extrapolated parts of LE (*f* ≤ 0.1) and CT (*f* ≥ 0.2) regimes. Reprinted with permission from Ref. [77]. Copyright 2012, Wiley-VCH.

Quite naturally, emission properties of luminescent materials are governed by their excited state characters. An LE state can have a large oscillator strength leading to high PLQY, while a CT state with reduced Δ*E*_ST_ may accelerate RISC. We can also perceive that the advantages from these two different ends can be combined in one molecule toward simultaneously achieving high EUE and high PLQY. Indeed, about a decade ago, Ma, Yang, and co-workers reported such a molecule with the D–A type construct, *N*,*N*-diphenyl-4′-(1-phenyl-1*H*-phenanthro[9,10-*d*]imidazol-2-yl)biphenyl-4-amine (TPA-PPI), consisting of a triphenylamine (TPA) donor and a 1,2-diphenyl-1*H*-phenanthro[9,10-*d*]imidazole (PPI) acceptor [77]. This molecule exhibited a large red-shift of 57 nm in emission spectra in going from a non-polar hexane solution to a highly polar acetonitrile one, indicating its CT character in the S_1_ state. However, it also displayed a very small solvatochromic shift in the low polarity regime, suggesting strong LE character in this case. These results add up to a conclusion that the S_1_-state character of this molecule switches from LE in low polar solvents to CT in highly polar ones, as shown in Figure 6. Density functional theory (DFT) analyses also suggested an “intercrossed CT and LE excited” state of the molecule, as the authors named it. The EUE of TPA-PPI was 28%, which is higher than the conventional statistical limit of 25%, showing that the intercrossed CT and LE state can indeed improve the efficiency of OLED devices.

A few years later, the same group reported another D–A type molecule, *N*,*N*-diphenyl-4-(9-phenylnaphtho-[2,3-*c*][1,2,5]thiadiazol-4-yl)aniline (TPA-NZP), consisting of a TPA donor and naphtho[2,3-*c*][1,2,5]thiadiazole (NZ) acceptor [51]. The device with TPA-NZP was reported to show an IQE of 14%. Considering that the PLQY of this molecule was 15%, a very high EUE of 93% was deduced. The excited state character also corresponded to an LE and CT combined state. Namely, TPA-NZP displayed similar solvatochromic shifts to those of TPA-PPI shown in Figure 6. In this work, they termed this type of excited state as a “hybridized local and charge-transfer” (HLCT) state. Besides the aforementioned state switching character depending on the solvent polarity, the HLCT state showed several additional characteristics. First, the fluorescence from this state displayed a single exponential decay without any delayed component. This single exponential lifetime evidenced that the LE and the CT characters are hybridized into one state, and that the excited species does not exist in two separated states. Second, the state character of T_1_ was LE, thus the energy level of T_1_ was significantly lower compared to the S_1_ level. Therefore, the traditional TADF mechanism, namely, the conventional RISC, could not work. In addition, in transient EL experiments, no TTA process was observed in devices with TPA-NZP (Figure 3a). Thus, they ascribed the improved efficiency to the RISC process between high-lying CT states (T*_n_* → S*_m_*), which they termed as hRISC to distinguish it from conventional RISC from T_1_.

**Figure 7:**
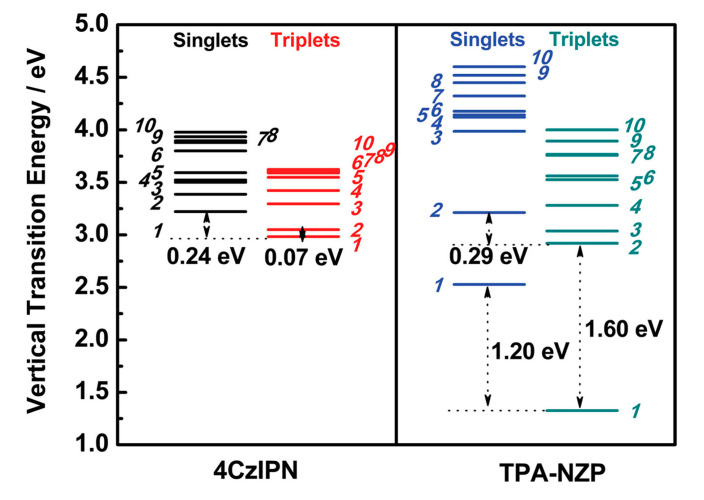
The 4CzIPN and TPA-NZP energy diagram of the first ten singlet and triplet excited states from TD-M06–2X calculations. Reprinted with permission from Ref. [84]. Copyright 2014, Wiley-VCH.

The group made further progress by comparing 4CzIPN, a typical TADF emitter, against TPA-NZP with the HLCT character [84]. For 4CzIPN, the lowest singlet and triplet excited states are CT states, and their energy gap was calculated to be as small as 0.24 eV, which is somewhat larger than the experimental value, 83 meV. Triplet excitons can convert to singlet by thermal energy with this small S_1_–T_1_ energy gap. In contrast, for TPA-NZP, both the S_1_ and the T_1_ states were of LE character, and their computed energy gap was as large as 1.20 eV. Thus, the S_1_–T_1_ RISC channel will hardly work. Instead, they reported that the S_2_ and the T_2_ states were CT states with quite a small energy gap of 0.29 eV. In addition, they explained that the large T_2_–T_1_ gap would suppress the internal conversion (IC) from T_2_ to T_1_, and that hRISC from T_2_ to S_2_ should in the end improve the efficiency of the OLED devices. The energy diagrams of the excited states for these two molecules are shown in Figure 7. Accordingly, they classified the two different spin conversions into “hot” and “cold” exciton processes.

As noted earlier, hot exciton materials can have several beneficial properties compared to other fully organic fluorescent materials. Because their S_1_ states have LE or HLCT characters, hot exciton emitters can have high luminescence efficiency with fast fluorescence, while TADF emitters can only have slow fluorescence caused by small transition dipoles with limited HOMO–LUMO overlaps, often leading to low PLQY. In addition, long-lived triplet excitons are significantly reduced due to fast hRISC. As the long-lived triplet excitons can break chemical bonds and/or decrease EUE by diverse exciton annihilation mechanisms, hot exciton materials can have both stability and efficiency. However, a large energy gap between triplet states to restrict IC becomes a rather limiting prerequisite. Based on the Fermi golden rule, transition rate is related to the state-to-state coupling and the energy gap [85], as given by

(3) $$k_{i\to f}=\frac{1}{\hbar^{2}}{\left|V_{fi}\right|}^{2}\int_{-\infty }^{\infty }dte^{i(E_{f}-E_{i})t/\hbar }$$

where *V* is the spin-orbit coupling in hRISC or the derivative coupling in IC processes. If hRISC takes place from T*_n_* to S*_m_*, the energy gap between T*_n_* and T*_n_*_−1_ must be large, and the derivative coupling should be small. Of course, the S*_m_*–T*_n_* gap needs to be small, and the corresponding SOC has to be large. Unlike the SOC, to which the so-called El-Sayed rule can apply [86], however, much less is known about how to control the derivative coupling. This imposes further difficulty in studying the hot exciton mechanism and designing new materials.

### 3.2. Evidence of Hot Exciton Mechanism

With the premise stated above, a series of hot exciton materials have been further reported [68,69,70,71,72,73,74,75,76]. Because directly observing the hRISC process is a formidable task, simple quantum chemical calculations have often been adopted to provide support for the mechanism. Given that, phenomenologically, the EUE of the device with a TTA emitter is limited to 62.5%, the EUE that exceeds this limit indicates the potential existence of hRISC, especially when there is no delayed component in the time-resolved emission. Moreover, a large S_1_–T_1_ energy gap of an emitter becomes strong evidence for excluding the possibility of TADF. On top of this, if there is a pair of singlet and triplet states with a small energy gap and large SOC, and if a large energy gap exists below the pinpointed triplet state, fast hRISC and slow triplet IC can be suggested. However, satisfying these conditions for eliminating alternative channels cannot constitute a definitive answer related to hRISC. Namely, even for cold exciton RISC that does not compete with IC, having a small S_1_–T_1_ gap and large SOC of an emitter does not necessarily establish success in finding an efficient TADF device; there is always competition between the radiative and nonradiative decay processes.

**Figure 8:**
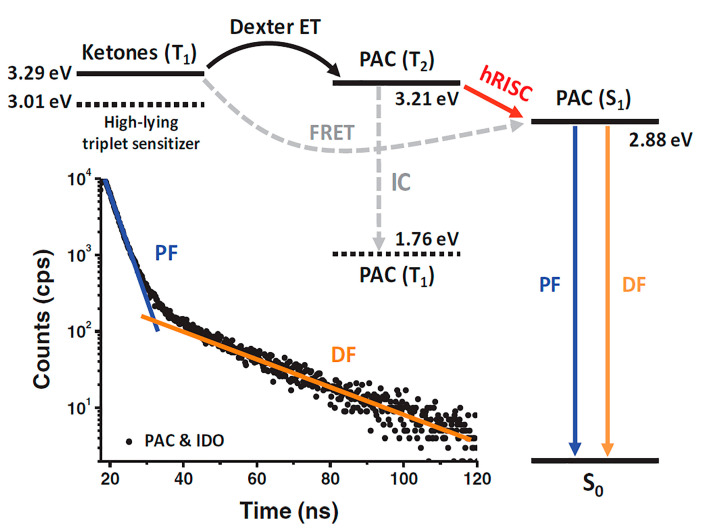
Diagram showing the energy transfer processes from ketones (IDO, BP) to PAC and the ensuing hRISC process in PAC. Solid arrows indicate dominant energy transfer processes, while dashed ones represent negligible processes. Reprinted with permission from Ref. [82]. Copyright 2019, Wiley-VCH.

Indeed, more rigorous or at least plausible explanations have been provided by utilizing energy-resolved triplet sensitizers. For example, Ma and co-workers reported 2-(4-(10-(3-(9*H*-carbazol-9-yl)phenyl)anthracen-9-yl)phenyl)-1-phenyl-1*H*-phenanthro[9,10-*d*]imidazole (PAC) [82] as a strong hot exciton emitter. They adopted platinum octaethylporphyrin (PtOEP) as a sensitizer to selectively initiate excitation to the T_1_ level of PAC as described and carried out transient absorption experiments to analyze the energy levels in the triplet manifold of PAC. Then, based on the T_2_ energy of PAC that they measured (3.21 eV), they employed two ketone sensitizers with different T_1_ energies: indanone (IDO, T_1_ = 3.29 eV) and benzophenone (BP, T_1_ = 3.01 eV) (Figure 8). Because the T_1_ energy of IDO is slightly higher than the T_2_ energy of PAC, in a solution with PAC and IDO, a delayed fluorescence component was detected, with the delay presumably coming from the sensitizing triplet–triplet energy transfer from IDO to PAC and the hRISC process between T_2_ and S_1_ of PAC. For a solution including both PAC and BP, on the other hand, such a sensitizing triplet energy transfer was not expected because the T_1_ energy of BP is lower than the T_2_ level of PAC. Therefore, no delayed fluorescence was observed. The chance of TTA involvement in PAC emission was additionally eliminated by adopting transient EL and MEL experiments. At present, utilizing a triplet sensitizer appears to provide the most direct evidence for supporting hot exciton processes, and thus several additional papers have adopted the same tactic to identify hot exciton mechanisms [87,88,89].

**Figure 9:**
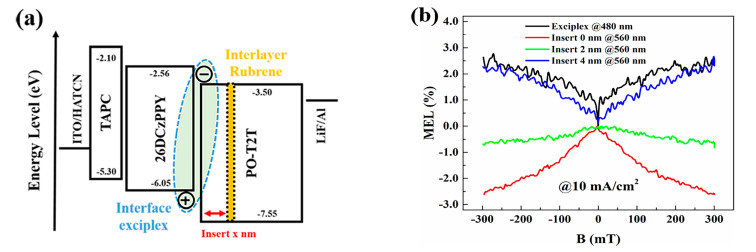
(**a**) Schematic diagram of the devices based on exciplex interface with the rubrene layer. (**b**) MEL responses of the devices with different distances involving the ultra−thin layer of rubrene. Reprinted with permission from Ref. [90]. Copyright 2022, American Institute of Physics.

As an additional tactic, magnetic field effects have further been examined to identify a hot exciton process. As described in Figure 3b, an ISC-dominant process involves a positive MEL curve, while an RISC-dominant process is accompanied by a negative MEL curve. Singlet fission and TTA processes display different but unique fingerprints. Xiong and co-workers reported different patterns in the devices with rubrene [52]. On the one hand, a non-doped rubrene device interestingly exhibited a MEL curve matching only singlet fission, presumably initiating from the emissive S_1_ state. On the other hand, a 4,4′-*N*,*N*′-dicarbazolebiphenyl (CBP)-rubrene device exhibited a negative MEL curve, indicating a RISC-dominant process in the device. With these, they suggested three components in the emission of rubrene: prompt fluorescence via FRET from CBP to rubrene, delayed fluorescence associated with hRISC from T_2_, which is directly sensitized via DET from the CBP triplet state, and TTA-mediated fluorescence after IC from T_2_ to T_1_. Later, Ma, Qiao, and co-workers made another doped rubrene device where an exciplex was formed by 2,6-bis(3-(9*H*-carbazol-9-yl)phenyl)pyridine and 2,4,6-tris[3-(triphenylphosphine)phenyl]-1,3,5-triazine [90]. They found that the MEL curves were positive in the exciplex emission region and negative in the rubrene emission region. These different MEL curves indicated that exciplex was ISC dominant (forming more triplets) and rubrene was RISC dominant (forming more singlets), implying that the increased emission by triplet harvesting must have involved the action of rubrene. They additionally designed a device that consisted of an exciplex interface and a thin rubrene layer with varying interface-to-layer distances as depicted in Figure 9. When the exciplex interface and the rubrene layer were close in distance, the MEL curve was negative, suggesting that RISC or hRISC was happening. Given that the rubrene was already confirmed to be acting for triplet harvesting, this indicated that DET from the exciplex interface to the rubrene layer was dominant. In contrast, when the distance was large, such that this DET was suppressed, the MEL curve changed into a positive pattern. These experimental data suggested that reaching rubrene emission involved a hot exciton channel with the triplet sensitizing host.

### 3.3. Computational Investigations on Hot Exciton Mechanism

Quantum chemical calculations and molecular dynamics simulations have been continually employed for analyzing hot exciton mechanisms. As mentioned earlier, such approaches can present insights into hot exciton mechanisms and help design new hot exciton emitters. Indeed, the capability of screening potential candidates will surely benefit from future studies. As mentioned in Section 3.1, hot exciton material should have a large energy gap and a small derivative coupling between T*_n_* and T*_n_*_−1_ and a small energy gap and a large SOC between T*_n_* and S*_m_*, assuming that the hRISC process occurs from T*_n_* to S*_m_* states. The energy gaps are more important [20] and easily controlled compared to the coupling, so adjusting the energy gap has been the main subject in computational studies.

Yang and co-workers investigated the state characters of hot exciton materials by calculating three D–A type molecules based on a triphenylamine (TPA) donor and three different acceptors: phenanthrene (PA), anthracene (AN), and acridine (AC), depicted in Figure 5 [91]. It is well accepted that the HLCT character is important for a hot exciton process. Out of the three molecules, TPA-AC, with the best singlet exciton generation, exhibited that its HLCT S_1_ state converted to a CT state with an increase in the twisting angle between the donor and the acceptor units, although the relationship between the good emission and such a state switching induced by the increased twisting angle [92,93,94,95] was not clearly unveiled. In addition, and quite expectedly, a triplet CT state was found to be very close in energy to the singlet HLCT state only in the best emitting molecule.

**Figure 10:**
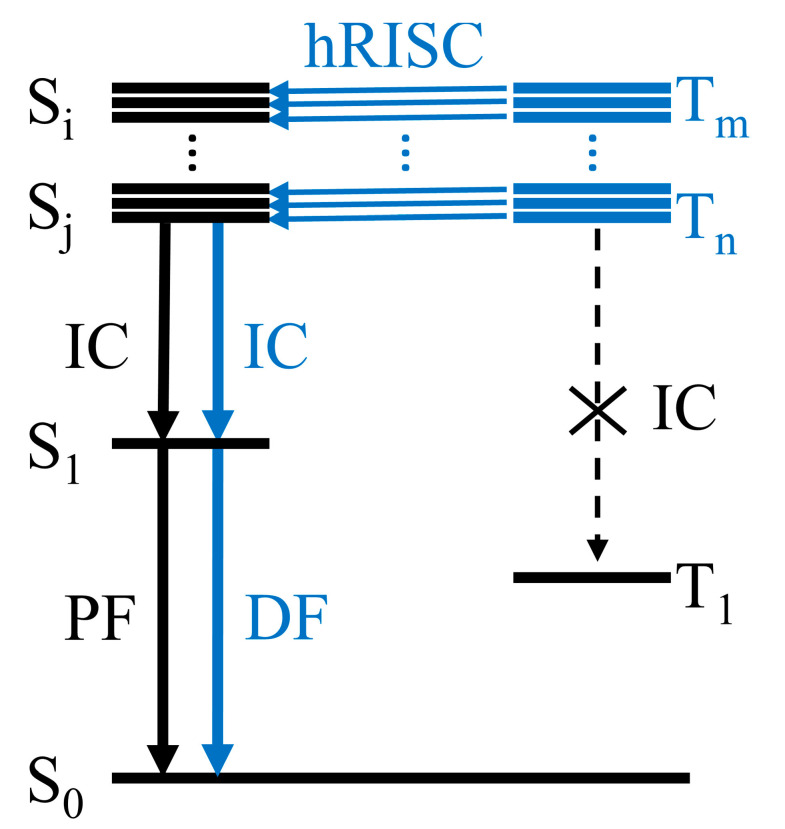
The concept of multiple hRISC channels. If the energy levels between several singlet and triplet excited states are similar, and if the SOC values between them are large enough, a multiple hRISC process can take place toward utilizing triplet excitons even with unrestricted IC.

In another study, Yang, Ma, and coworkers investigated para- and meta-linked forms of TPA-PPI (in Figure 5) and their derivatives [96]. This study was based on the previous work in which mTPA-PPI had improved color purity while maintaining efficiency comparable to that of TPA-PPI [78]. Because the PPI unit is widely used for hot exciton materials [97,98,99,100,101], analyzing the character of PPI-based molecules will be fruitful for understanding hRISC characters. The meta-linked forms generally displayed reduced structural differences between the S_1_ and the S_0_ optimized conformations, decreasing vibronic coupling induced by conformational relaxation, which expectedly resulted in a narrow emission. However, the meta-linked forms showed decreased CT characters in the S_1_ state leading to a larger energy gap for the hRISC channel. Accidentally, however, there were high-lying singlet and triplet states that actually had better energy alignment and more preferable SOC values for efficient hRISC. They stated that this aspect opened a possibility that multiple hRISC channels could act in the meta-linked forms as described in Figure 10. This would in turn preserve emission efficiencies for these molecules in comparison with their para-counterparts. In addition, small structural changes in meta-derivatives could increase the LE character in the HLCT state and improve color purity without sacrificing efficiency.

Condensed phase QM/MM simulations may help explain restricted IC in hot exciton materials [102,103]. In the condensed phase, low-frequency twisting motions are suppressed and conformational relaxation between the ground state and the excited state is reduced, likely due to the crowding effect by the surrounding molecules. This will reduce the Huang-Rhys factor and the reorganization energy. Subsequently, IC from S_1_ to S_0_ will be slowed down and the fluorescence efficiency can be improved. Therefore, the aggregation-induced emission mechanism [104,105,106] can surely be utilized for designing hot exciton materials.

As mentioned above, screening hot exciton candidates will be a fruitful task in designing new materials. Fumanal, Meng, and co-workers calculated 234 donor–acceptor–donor type molecules based on 13 donor groups and 18 acceptor groups [107]. They determined whether a molecule could function as a hot exciton emitter based on three criteria: the injection energy, the energy gap between any triplet states to restrict IC, and the energy gap between singlet and triplet states for efficient hRISC. With these three criteria, only 36 out of the 234 molecules were determined to be candidates for hot exciton emitters. To further confirm the potential of hRISC, they calculated SOCs between singlet and triplet states that were close in energy. Among the 36 D–A–D systems, 31 molecules exhibited large enough SOC toward potential hRISC with the remaining five possessing small SOC values below 0.1 cm^−1^. Several other papers also calculated energy levels and SOC values in diverse systems, with the purpose of helping design new hot exciton materials [108,109,110].

### 3.4. Hot Exciton Material Design

In fact, numerous attempts to newly design and synthesize hot exciton materials have been reported. While it is yet somewhat ambiguous or even difficult to define definitive design rules, a number of strategies have been adopted, especially by combining fragments, such as D–π–A [111], D–A–D [68], A–π–A [112], and D–π–A–π–D [113] with additional potential decorations on the linkers with triple bonds [114]. In this part, we will review some cases with insights that one can apply to designing hot exciton materials in general.

The main issue related to hot exciton molecules is whether we can restrict unavoidable IC in the triplet manifold. As most hRISC processes initiate from T_2_ states, increasing the T_2_–T_1_ energy gap will be a good strategy. When there is a core fragment with a given T_1_ energy, if we introduce a substituent with a much higher T_1_ energy of its own, the T_2_ energy of the whole molecule will be close to the T_1_ energy of the added substituent [80,115] as long as the T_2_ energy of the original core part is even higher. This hypothesis was proven by comparing two D–A–D molecules that combined a naphtho[2,3-*d*][1,2,3]triazole (NMZ) core and either naphthalene (Np) or anthracene (An) substituents. The T_1_ energy of Np was higher than the T_1_ energy of An, and, subsequently, Np–NMZ–Np had a larger T_2_–T_1_ energy gap than An–NMZ–An [80]. In addition, for a D–A type molecule, the electronic coupling between LE T_1_ and CT T_2_ states correlates with the electron exchange between the donor and the acceptor fragments. Therefore, a twisted conformation will restrict the IC between the two states. If hRISC takes place by involving these states, inducing a twisted conformation may be a good strategy [116].

In addition to structural modifications on emitters, the doping effect in a device may also be important. For example, rubrene and 10,10′-diphenyl-9,9′-bianthracene did not exhibit any hot exciton processes in neat films of non-doped devices. However, the device efficiency was significantly increased when they were used as dopants in a CBP host matrix [52,81]. For example, when rubrene was doped in a CBP host, the maximum EQE was 5.22%, while it was 0.55% in neat film. Furthermore, when hot exciton molecules were used as the host, the device performance was also notably enhanced [83]. The maximum EQE values of the non-doped devices with PAC or 4⁗-(diphenylamino)-2″,5″-diphenyl-[1,1″:4′,1″:4″,1‴:4‴,1⁗-quinquephenyl]-4-carbonitrile (TPB-PAPC) in Figure 5 were 10.2% and 6.0%, respectively. However, upon adopting a traditional fluorescent emitter, *N*,*N*′-bis(3-methylphenyl)-*N*,*N*′-bis[3-(9-phenyl-9*H*-fluoren-9-yl)phenyl]-pyrene-1,6-diamine (BD), the maximum EQE increased to 17.4% and 9.0%, respectively. [83]. It was explained that the singlet exciton of PAC and TPB-PAPC transferred to BD by FRET, and that this depletion of the singlet population accelerated the hRISC process in the host. In addition, the TTA process in the device with the PAC host was somehow inhibited compared to that in a non-doped device, likely because the accelerated hRISC process in the PAC host also suppressed IC to T_1_, subsequently diminishing the TTA source. These results indicate that not only designing new emitter molecules but also matching them with appropriate host or dopant molecules will be important.

### 3.5. Future Computational Prospects on Hot Exciton Materials

Unlike RISC in TADF molecules, the hRISC process takes place in high-lying excited states. Calculating the properties of such states tends to be computationally expensive compared to calculating the ground state or even the lowest excited state properties. For this reason, existing studies have usually adopted approximations for reducing computational costs. Here, we will discuss what approximations people have used and how we can improve fidelity in future considerations.

**Figure 11:**
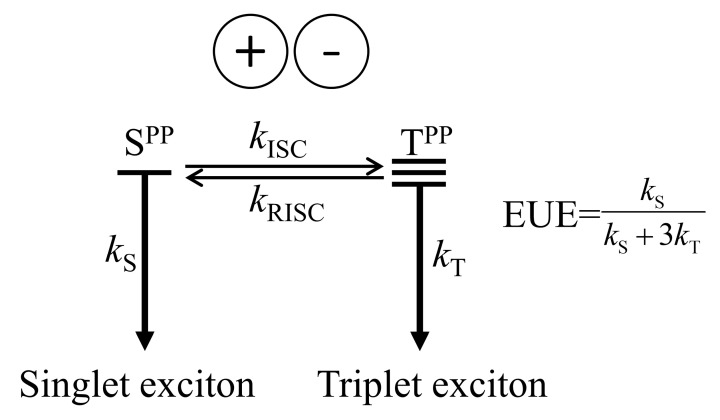
Charge recombination processes in OLED devices. If the spin conversion process between singlet and triplet polaron pairs (PP) is much faster than the charge recombination process (*k*_ISC_, *k*_RISC_ >> *k*_S_, *k*_T_), the device’s EUE will depend on the charge recombination rates.

Currently, computational studies are struggling to explain the anti-Kasha characteristics, namely, the restricted IC in the triplet manifold. Although the condensed phase effect suppresses molecular motions and reduces the IC rate [102,103], to the best of our knowledge, computational evidence that explicitly shows that the hRISC rate is actually faster than or at least comparable to the IC rate have not been reported. Several papers reported predictions on the hRISC rates with the help of the thermal vibration correlation function (TVCF) formalism [117]. However, the IC rates were either not really compared to [102,118,119] or actually shown to be much faster than the hRISC rate [120]. Nevertheless, solid experimental evidence exists to show that the hot exciton materials bear preferable properties that cannot be explained through TADF and/or TTA mechanisms. This may be related to the fact that the TVCF formalism is based on harmonic oscillator models, which becomes unreliable when the conformational relaxation between the two involved states is large [117]. If the limitation is due to the anharmonicity, given that its remedy has been recently reported [121], it will be interesting to see how the improved formalism behaves in the predictions on hRISC systems. In addition, geometric displacements expressed in Cartesian are not appropriate for describing twisting motions, which will likely be important in many donor–acceptor and related molecular constructs. For this shortcoming, adopting internal displacements [122,123] may become a good alternative. Nevertheless, twisting motions often induce severely nonharmonic potential energy changes. Therefore, more appropriate formalisms for handling large structure differences, albeit challenging to develop, will be needed. On the flip side, there remains a possibility that a new mechanism could be suggested to explain the overall dynamics: for example, an event led by multiple hRISC channels [96,109]. In addition, the charge recombination processes experienced by polaron pairs in the early stage of recombination is another overlooked process. Indeed, if the charge recombination rate depends on spin multiplicities, the conventional rule on the singlet-triplet ratio can be violated (Figure 11) [50,124]. Testing on these hypothetical scenarios will surely require us to adopt various quantum chemical calculations and diverse simulation studies.

When computationally deducing hot exciton processes, one should conduct geometry optimizations in high-lying states before calculating state energies and SOC values. However, such geometry optimizations require greater resources and even become more cumbersome as we walk up higher in state indexes, mainly due to the notorious state-switching issue [125,126]. Likely for this reason, some studies have only used vertical excitation energies to evaluate the energy gap [96,107], which will inevitably impair the reliability. We should also note that Δ*E*_ST_ is not the best indicator for judging the plausibility of ISC/RISC processes. Indeed, with the TVCF formalism, it was reported that the Marcus-type barrier provided better agreement than simple Δ*E*_ST_ in TADF systems [127]. The Marcus-type barrier contains the reorganization energy associated with the state switch, and this aspect also suggests the importance of employing fully optimized geometries.

## 4. Concluding Remarks

TADF, TTA, and hot exciton mechanisms are representative triplet harvesting processes that enable us to overcome the limitation of the spin statistics in OLED. TADF emitters can yield ~100% IQE, but long-lived high energy triplet excitons reduce the stability of emitters. Because the T_1_ energies of TTA emitters are only halves of the S_1_ energies, they are free from such high energy exciton issues, but the IQE is limited to 62.5% at best. In addition, high densities of triplet excitons make them vulnerable to undesirable exciton quenching processes. The hot exciton process can be free from such pitfalls of long-lived triplet excitons. It feeds singlet states directly from high-lying triplet excited state(s), and in principle, the hot exciton emitters may reach ~100% of IQE. Adapting the hot exciton concept can expand the candidates in designing fluorescent emitters for OLED applications. For example, recently, even MR-TADF emitters with hRISC processes have been reported [128,129,130,131]. This means that we need to expand our focal points beyond the conventional S_1_-T_1_ energy gap. Due to the relatively short history and added physical complexity, fundamental research on hot exciton processes from high-lying excited states is still lacking. Probably, complicating high-lying excited states with the unconventional anti-Kasha characteristics may be the main targets for the study of hot exciton mechanisms.

In this regard, the main difficulty in studying hot exciton processes lies in finding the relevant excited state. RISC in a TADF molecule takes place between S_1_ and T_1_, and we definitely know where to focus in revealing the state properties. In contrast, hRISC in hot exciton materials can involve any state within the triplet manifold created after the charge recombination and any singlet states with similar energies. Even when an emitter by itself does not exhibit the characteristics, the hot exciton characters may still show in a device with a proper host that can act as a triplet sensitizer [52,81]. Despite the difficulty, there are continuing studies that are analyzing the excited state characters of hot exciton materials.

The study of the hot exciton mechanism is challenging in both theory and experiment. Generally, the nonradiative decay lifetimes of fluorescent emitters are at best in the nanosecond regime. The energy gaps between energetically neighboring triplet states in hot exciton molecules tend to be much smaller than the visible gap, and, therefore, the connecting nonradiative decay (IC) lifetimes will likely be shorter than nanoseconds. On the other hand, calculated hRISC lifetimes are often slower than sub-nanoseconds or even microseconds. It is not clearly known yet how this relatively fast IC can be avoided to promote hRISC, but high-efficiency emitters which cannot be explained by TADF or TTA are being continually reported. Thus, clear elucidation on the details of the hot exciton processes will be able to greatly promote the construction of design strategies of highly efficient emitters in the future. As discussed in Section 3.5, multiple hRISC and/or competing charge recombination processes can hypothetically explain how hRISC takes place despite fast IC. For designing OLED materials with computational means, the ability of calculating various transition rate coefficients can play an important role for both radiative and nonradiative decays, as the calculation can directly lead to property prediction such as the luminescence efficiency. Indeed, numerous studies have been reported for such calculations [119,132,133,134,135], and similar studies may prove their utility, especially toward testing the hypotheses. Provided that there is still some room for practical improvements for the related formalisms as mentioned in Section 3.5, we expect that future methodology developments and computational studies based on them will provide valuable insights for designing hot exciton materials.

## Figures and Tables

**Table 1:** Photophysical properties of representative hot exciton materials.

Compounds	λ ^a^ (nm)	PLQY (%)	IQE (%)	EUE (%)	EQE_max_ (%)	CIE (x, y)	Ref.
TPA-PPI	438	90	25	28	5.02	0.15, 0.11	[77]
TPA-NZP	632	15	14	93	2.8	0.67, 0.32	[51]
mTPA-PPI	404	35	17	48	3.33	0.161, 0.049	[78]
TPA-PA	418	70	10	14	2.0	0.15, 0.07	[79]
TPA-AN	460	50	15	30	3.0	0.15, 0.23	[79]
TPA-AC	504	35	16	46	3.2	0.20, 0.51	[79]
2NpNMZ ^b^	502	61.0	17	29	5.39	0.19, 0.51	[80]
2AnNMZ ^b^	526	37.8	13	35	4.50	0.32, 0.62	[80]
PPBA ^b^	440	80	-	-	11	-	[81]
PAC	458	48	-	-	10.5	0.15, 0.13	[82]
TPB-PAPC	450	65	30	46	6.0	0.14, 0.07	[83]

^a^ Emission wavelength. ^b^ Properties of doped devices.

## Data Availability

Not applicable.
